# Pulmonary Veno-Occlusive Disease in Systemic Sclerosis: A Scoping Review of the Literature

**DOI:** 10.3390/jcdd13100506

**Published:** 2026-10-08

**Authors:** Francesco Carta, Beatrice Moccaldi, Giulia Famoso, Chiara Giraudo, Anna Cuberli, Marco Binda, Andrea Benini, Martina Perazzolo Marra, Roberta Ramonda, Laura De Michieli, Elisabetta Zanatta

**Affiliations:** 1 Rheumatology Unit, Department of Medicine-DIMED, Padova University Hospital, 35128 Padova, Italy; francesco.carta@aopd.veneto.it (F.C.); beatrice.moccaldi@aopd.veneto.it (B.M.); andrea.benini@aopd.veneto.it (A.B.);; 2 Cardiology Unit, Department of Cardiac, Thoracic, Vascular Sciences and Public Health, Padova University Hospital, 35128 Padova, Italymartina.perazzolomarra@unipd.it (M.P.M.);; 3 Unit of Advanced Clinical and Translational Imaging, Department of Cardiac, Thoracic, Vascular Sciences and Public Health-DCTV, University of Padova, 35128 Padova, Italy; chiara.giraudo@unipd.it; 4 Department of Surgery, Oncology and Gastroenterology (DiSCOG), Padova University Hospital, 35128 Padova, Italy; 5 Department of Cardiac, Thoracic, Vascular Sciences and Public Health, Padova University Hospital, 35128 Padova, Italy

**Keywords:** pulmonary veno-occlusive disease, pulmonary hypertension, systemic sclerosis, connective tissue disease

## Abstract

**Background**: Patients with systemic sclerosis (SSc) may be affected by pulmonary veno-occlusive disease (PVOD), a poorly characterized form of group I pulmonary arterial hypertension (PAH). This scoping review aimed to systematically map the literature regarding SSc-PVOD to identify key clinical features, knowledge gaps, and future research areas. **Methods**: A systematic literature search was conducted (January 1970 to May 2026) across PubMed, Scopus, and Cochrane databases following the PRISMA-ScR guidelines. We included adult patients with right heart catheterization (RHC)-confirmed SSc-PAH and a concurrent/subsequent diagnosis of PVOD established via pulmonary edema following vasodilators, highly suggestive chest HRCT findings, or histopathology. **Results**: A total of 20 articles were included, encompassing 63 SSc-PVOD patients (87.2% females). Chest HRCT revealed ≥2 highly suggestive abnormalities (centrilobular ground-glass opacities, interlobular septal thickening, mediastinal lymphadenopathy) in 46/54 patients. Almost all patients had mPAP > 35 mmHg at diagnosis, and 12/25 had DLCO < 30%. Lung tissue was obtained in 13 patients (11 post-mortem); predominant histological findings were venous luminal narrowing (11/13), arteriole fibrosis (9/13), and thickened interalveolar septae (9/13). Notably, 20 out of 24 patients treated with pulmonary vasodilators developed pulmonary edema. Overall, two patients underwent lung transplantation, and 33 (52%) died. **Conclusions**: SSc-PVOD mimics classic SSc-PAH at RHC but carries a high risk of PAH-treatment-induced pulmonary edema. Early non-invasive recognition is vital to avoid drug-related harm and prompt transplant referral. Novel anti-remodeling therapies should be explored.

## 1. Introduction

Systemic sclerosis (SSc) is a complex autoimmune connective tissue disease (CTD) characterized by immune dysregulation, widespread microvascular damage, and progressive tissue fibrosis. Among the various complications of SSc, pulmonary arterial hypertension (PAH) represents the leading cause of morbidity and mortality after interstitial lung disease (ILD), affecting up to 8–12% of patients [1]. Pulmonary veno-occlusive disease (PVOD) and pulmonary capillary hemangiomatosis (PCH)—now grouped under Group 1 in the updated World Health Organization (WHO) classification [2]—represent a rare, highly challenging, and underrecognized form of SSc-associated pulmonary vascular involvement [3].

PVOD is pathologically defined by fibrous, obliterative remodeling of the small post-capillary pulmonary veins and venules, frequently accompanied by secondary capillary engorgement or angioproliferation (PCH). Histopathological, clinical, and genetic analyses demonstrate that PVOD and PCH represent slightly different morphological expressions of the exact same underlying pathological phenomenon, with PCH being fully assimilable to PVOD in terms of clinical course, diagnostic indicators, and management [4,5,6]. Therefore, throughout the remainder of this review, the term PVOD is utilized to collectively encompass both entities [7]. Such pathological changes occurring in the pulmonary venules lead to pulmonary capillary congestion, fluid accumulation and localized interstitial edema, resulting in characteristic and highly suggestive thoracic radiological findings on high-resolution computed tomography (HRCT)—centrilobular ground-glass opacities, smooth interlobular septal thickening and mediastinal lymphadenopathy [8]. Clinically and hemodynamically, SSc-PVOD closely mimics classic SSc-PAH, presenting with exertional dyspnea, progressive right-sided heart failure and pre-capillary pulmonary hypertension on right heart catheterization (RHC) [9]. However, making an accurate differential diagnosis is of paramount clinical importance. Administering PAH-targeted vasodilator therapies (such as endothelin receptor antagonists—ERAs, phosphodiesterase-5 inhibitors—PDE5is, or prostacyclin analogs) to patients with underlying PVOD may abruptly increase blood flow into an inextensible capillary bed, elevating transcapillary hydrostatic pressure and inducing life-threatening acute pulmonary edema [8].

Despite its severe prognostic implications, the true prevalence and optimal diagnostic algorithms for SSc-PVOD remain poorly defined. Current guidelines by the European Society of Cardiology and the European Respiratory Society (ESC/ERS) recommend annual screening for PAH in SSc using transthoracic echocardiography, pulmonary function tests (PFTs), and cardiac biomarkers [2]. However, distinguishing PVOD from classical SSc-PAH remains challenging because of their substantial clinical and hemodynamic overlap [10]. In this context, the identification and characterization of clinical, functional, radiological and hemodynamic features that may raise suspicion of PVOD is particularly relevant. However, these aspects have not been systematically characterized in the literature, as the available evidence mainly consists of small case series and case reports, with substantial heterogeneity in reported data.

Therefore, we conducted a scoping review according to the PRISMA-ScR guidelines to systematically map the existing literature on SSc-PVOD [11], characterize the epidemiological, serological, functional, hemodynamic, and radiological phenotype of SSc-PVOD, and highlight existing knowledge gaps, which may guide the design of future prospective studies.

## 2. Materials and Methods

### 2.1. Identification of Studies and Literature Search Strategy

The review was conducted in accordance with the Preferred Reporting Items for Systematic Reviews and Meta-Analyses extension for Scoping Reviews (PRISMA-ScR) guidelines [12] (Supplementary Material S1). The review protocol was established *a priori* and subsequently registered on the Open Science Framework (OSF) following study selection and data extraction (registration DOI: 10.17605/OSF.IO/TR8H5). The research question was formulated using the Population, Concept, and Context (PCC) framework as follows: adult patients with pre-capillary SSc-PAH confirmed at RHC with a concurrent or subsequent diagnosis of PVOD or PCH. A comprehensive literature search was executed across electronic databases including PubMed, Scopus, and the Cochrane Library for articles published between January 1970 and May 2026, with the final search run on 20 May 2026. The search strategy combined Medical Subject Headings (MeSH) and free-text terms searched within the title or abstract of articles using Boolean operators (AND, OR) adapted for each database. Keywords targeting the concept included “pulmonary veno-occlusive disease”, “PVOD”, “pulmonary capillary hemangiomatosis”, “PCH”, “pulmonary venous involvement”, “pulmonary venous hypertension”, “postcapillary vasculopathy”, and “venous remodeling”. Keywords targeting the population encompassed “systemic sclerosis”, “SSc”, “scleroderma”, “CREST syndrome”, “limited cutaneous systemic sclerosis”, “lcSSc”, “diffuse cutaneous systemic sclerosis”, “dcSSc”, “connective tissue disease”, and “CTD” (Supplementary Material S2).

### 2.2. Eligibility Criteria and Study Selection

Inclusion criteria comprised:

− Adult SSc patients presenting with PAH confirmed by RHC, defined according to the updated criteria as a mean pulmonary arterial pressure (mPAP) > 20 mmHg, a pulmonary arterial wedge pressure (PAWP) ≤ 15 mmHg, and a pulmonary vascular resistance (PVR) > 2.0 Wood Units [2].
− A concurrent or subsequent diagnosis of PVOD or PCH established via at least one of the following:
  ○ Histopathological confirmation (showing venous luminal narrowing, intimal fibrosis, or thickened interalveolar septa).
  ○ Highly suggestive thoracic HRCT findings (centrilobular ground-glass opacities, smooth interlobular septal thickening, or mediastinal lymphadenopathy).
  ○ Clinical/ex juvantibus diagnosis characterized by sudden respiratory deterioration or acute pulmonary edema following vasodilator initiation [13].
− Availability of demographic, clinical, hemodynamic, or radiological data.
− Original research articles, including observational cohort studies, case–control studies, case series, and case reports.

### 2.3. Exclusion Criteria

− Presence of interstitial lung disease (ILD) or significant parenchymal fibrotic changes on HRCT (defined as >20% extent of parenchymal fibrosis according to the Goh et al. staging criteria) [14].
− Moderately severe to very severe obstructive pattern on PFT: Forced expiratory volume in 1 s (FEV1) < 60% with FEV1/forced vital capacity (FEV1/FVC) <70% of predicted.
− Restrictive pattern on PFT: total lung capacity inferior to lower limit of normal (TLC < LLN).
− Presence of other non-Group 1 forms of pulmonary hypertension [2].
− Pediatric or juvenile SSc cases.
− Preclinical experimental studies.
− Abstracts without accessible full texts or articles in languages other than English.

**Figure 1:**
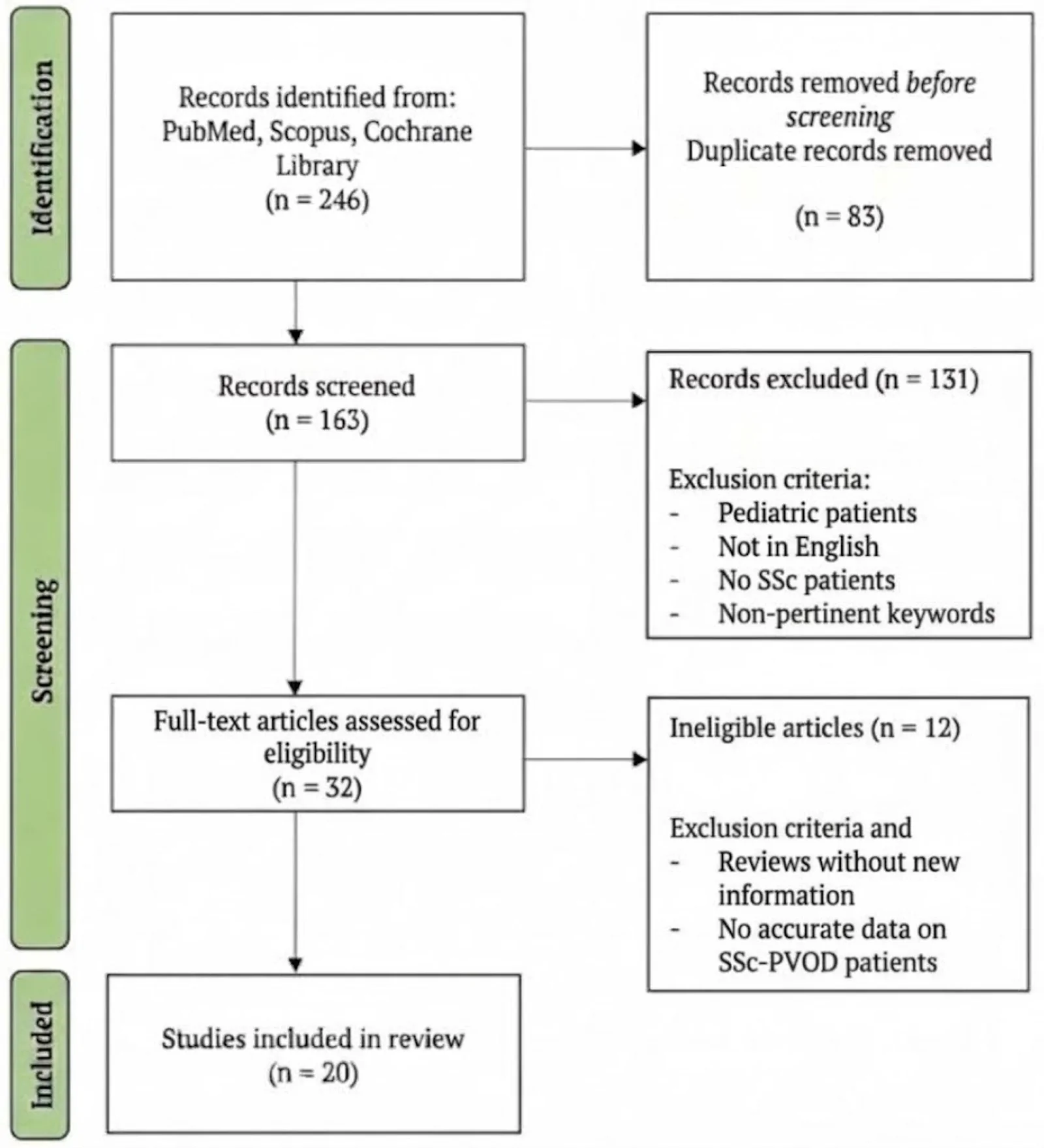
PRISMA flow-chart illustrating the updated phases of records identification, screening, and inclusion for the scoping review.

Search results were uploaded to the Rayyan web-based screening platform for duplicate removal and title/abstract screening [15]. The initial search yielded 246 records. Following the removal of 83 duplicates, 163 unique records were screened by title and abstract, resulting in the exclusion of 131 non-pertinent studies. The remaining 32 full-text articles were rigorously evaluated against the eligibility criteria, leading to the exclusion of 12 studies. Ultimately, 20 articles met all inclusion criteria and were incorporated into the scoping review (Figure 1). The literature search, study screening, and eligibility assessments were performed independently by two reviewers (FC, BM), with any discrepancies resolved through consensus. Data extraction was conducted using a standardized data abstraction form. Extracted variables included demographic parameters (age, sex); SSc disease characteristics (cutaneous subtype, autoantibody status); HRCT morphological features (ground-glass opacities, centrilobular/diffuse/patchy patterns, mosaic perfusion, interlobular septal thickening, mediastinal lymphadenopathy, pleural and pericardial effusions, pulmonary artery dilatation, and the number of cardinal CT signs per patient); hemodynamic parameters from RHC (mPAP, PAWP, cardiac output, PVR); PFTs, including FEV1%, FVC in liters and %, FEV1/FVC (%), TLC%, and diffusing capacity of the lung for carbon monoxide (DLCO%); 6 min walk test (6MWT); the use of PAH-specific treatments and clinical outcomes including pulmonary edema, acute right heart failure, lung transplantation, and all-cause mortality.

### 2.4. Data Synthesis and Qualitative Analysis

The selected studies were characterized by heterogeneous study designs ranging from single-case reports and small retrospective case series to observational cohorts and registry data. Due to the inability to obtain individual patient data (IPD) from the latter type of studies, which primarily reported group-level summary statistics, individual-level correlative analyses (e.g., cross-evaluating associations between DLCO drop, HRCT signs, and pulmonary edema risk) could not be performed. Hence, the data synthesis was structured as a comprehensive narrative synthesis and thematic mapping. Extracted metrics were categorized into defined clinical, functional, hemodynamic, radiological, and outcome domains. Continuous variables from original studies were summarized using descriptive statistics, presented as means ± standard deviations or medians with ranges/interquartile ranges as originally reported, while categorical variables were expressed as absolute counts and percentages.

## 3. Results

### 3.1. Study Selection and PVOD Diagnostic Criteria Application

The systematic literature search identified 20 eligible studies with a total cumulative cohort of 63 patients diagnosed with SSc-PVOD or PCH. As detailed in Table 1, the included papers comprised a heterogeneous mix of study designs: 12 individual case reports [9,16,17,18,19,20,21,22,23,24,25,26]; 2 case series [27,28]; 1 retrospective case–control study [29]; 3 retrospective cohort studies [10,30,31]; and 2 combined retrospective cohorts with autoptic case series [8,32].

The diagnostic criteria applied across these 20 studies to establish PVOD/PCH were categorized into three primary domains:

− Histological confirmation documented by eight studies [17,20,23,25,27,29,30,32];
− Presence of ≥2 cardinal chest HRCT finding (centrilobular ground-glass opacities, mediastinal lymphadenopathy, interlobular septal thickening) documented by 12 studies [8,9,10,16,18,19,23,24,26,27,28,31];
− Acute pulmonary edema following vasodilator therapy documented by nine studies [9,16,18,19,21,22,24,25,30].

### 3.2. Epidemiological, Clinical, and Autoantibody Profile

Across the cumulative population of 63 patients, sex distribution was specified in 47 subjects, revealing a clear female predominance of 41 females (87.2%) compared to 6 males (12.8%), whereas the remaining 16 patients belonging to the cohort by Günther et al. (2012) lacked individual sex characterization in the extracted data [8] (Table 2). Age at presentation or evaluation spanned between the fifth and seventh decades of life, with specific reported cohort means of 59 ± 9 years in Connolly et al. (2017), 69 ± 6 years in Daraban et al. (2015), and 66 ± 8 years in Dorfmüller et al. (2007) [10,16,29]. Individual point ages from single-case reports further mirrored this distribution, ranging from 45 to 75 years [24,26,27].

Information regarding the SSc cutaneous subtype was available in 43 patients, with the most common being the limited cutaneous variant (lcSSc), present in 40 cases (93.0%), while diffuse cutaneous SSc (dcSSc) was documented in only 3 cases (7.0%) all belonging to Günther et al., 2012 [8]. In the remaining 20 patients, the subtype was either not reported or indicated as 0/0. Serological autoantibody profiling, fully characterized across 14 patients, revealed a strong association with anti-centromere antibodies (ACAs), which were positive in 13 subjects (92.8%). Conversely, anti-topoisomerase I (Anti-Scl70) and Anti-PM-Scl antibodies were universally absent across all 14 characterized cases. Isolated positivity was documented for anti-fibrillarin, anti-RNA polymerase III, and anti-Th/To in single cases within the cohort by Connolly et al. (2017), as well as anti-Ro/SSA in Kyono et al. (2020) and anti-U1-RNP in Jeevagan (2010) [10,20,21]. Notably, Kyono et al. (2020) described a unique clinical course where PVOD developed in a female patient with a prior 8-year history of ACA-positive primary Sjögren’s syndrome who transitioned to lcSSc prior to the onset of severe pulmonary hypertension [21].

### 3.3. Thoracic HRCT Imaging Parameters

Thoracic CT or HRCT imaging findings were individually detailed in 19 patients (Table 3). Ground-glass opacities (GGOs) were documented in 9 of these 19 reported scans (47.4%), displaying diverse distribution patterns that included centrilobular GGOs in one patient, diffuse GGOs in four patients and patchy GGOs in two patients. Furthermore, interlobular septal thickening was identified in five scans (26.3%), while mediastinal lymphadenopathy was observed in seven scans (36.8%). Vascular and extra-parenchymal abnormalities included pulmonary artery (PA) dilatation in eight cases (42.1%) and pleural effusion in four cases (21.1%), whereas mosaic perfusion and parenchymal fibrosis were each observed in single isolated cases.

When analyzing the cumulative co-occurrence of cardinal HRCT signs per patient across the entire characterized population of 54 subjects, 45 patients (83.3%) harbored ≥2 features. In contrast, despite developing acute pulmonary edema after PAH treatment or having histological diagnosis of PVOD, five patients (9.3%) exhibited only one cardinal sign and three patients (5.6%) presented no classical signs. Lastly, a single patient manifested all three cardinal features [16].

### 3.4. Hemodynamic Profile at Right Heart Catheterization

Hemodynamic measurements at RHC were available across 57 patients, consistently confirming a severe pre-capillary pulmonary hypertension profile (Table 4). The mPAP spanned from a minimum of 26 mmHg to a maximum of 72 mmHg [21,22]. Cohort mean mPAP values were recorded at 38 ± 8 mmHg in Connolly et al. (2017), 51 ± 3 mmHg in Dorfmüller et al. (2007), 48 ± 10 mmHg in Günther et al. (2012), 38 ± 1 mmHg in Nakamura et al. (2023), and 49.1 ± 9.4 mmHg in Smith et al. (2024) [8,10,28,29,31].

PAWP, reported in 52 patients, remained strictly ≤15 mmHg across the entire literature, ranging between 3 mmHg and 14 mmHg. Cardiac output (CO), evaluated in 44 patients, showed means of 4.6 ± 1.3 L/min in Günther et al. (2012), 3.7 ± 0.5 L/min in Nakamura et al. (2023), and 3.9 ± 1.8 L/min in Smith et al. (2024), alongside point values ranging from 2.1 L/min to 8.0 L/min [8,28,31]. PVR (N = 54) was substantially elevated across studies, with aggregated means of 6.44 ± 2.35 WU in Connolly et al. (2017), 27.2 ± 10.8 WU in Dorfmüller et al. (2007), 9.8 ± 4.2 WU in Günther et al. (2012), 9.0 ± 1.3 WU in Nakamura et al. (2023), and 12.0 WU in Smith et al. (2024), accompanied by single-case values reaching up to 20.0 WU [8,10,28,29,31,32].

### 3.5. Major Complications, Treatments, and All-Cause Mortality

Data on PAH-specific therapy was very limited. Overall, the use of vasodilators is reported for 24 patients, while information of specific pharmacological class is available for only 11. Of those, three patients underwent combination therapy with an endothelin receptor antagonist (ERA) and a phosphodiesterase-5 inhibitor (PDE5i), two monotherapy with ERA and two with PDE5i [9,18,25,26]. One patient [25] was treated with triple therapy with an ERA, a PDE5i and parenteral prostanoids. Two patients [19,22] received parenteral prostanoid monotherapy, while in one patient [30] parenteral prostanoids were administered in combination with an ERA.

Information regarding the administration of PAH-specific vasodilators was documented in only 24 of the 63 included patients. Notably, 21 of these 24 treated patients (87.5%) experienced major treatment-related complications following the initiation or dose escalation of targeted PAH vasodilators (Table 5); among them, acute pulmonary edema was by far the predominant adverse event, occurring in 20 patients (95.2% of complications; 31.7% of the total cumulative cohort). These pulmonary edema events were distributed across multiple studies, including 2/7 in Connolly et al. (2017), 2/4 in Dorfmüller et al. (2007), 8/16 in Günther et al. (2012), and 1/4 in Johnson et al. (2006), as well as in single-case reports [8,10,27,29]. Acute right heart failure was recorded as a major complication in three additional patients. Among patients with available data on vasodilator treatment (N = 11), eight patients developed treatment-related complications. The remaining three patients, who were treated with vasodilators without relevant side effects, were treated with combination ERA + PDE5i, ERA monotherapy and PDE5i monotherapy, respectively [17,21,26]. Lung transplantation was successfully performed in only two patients (3.2% of the total cohort), whereas five patients (7.9%) were placed on the active transplant waitlist but died prior to donor organ availability [17,29].

## 4. Discussion

PVOD represents a rare and life-threatening form of PAH that can occur in patients with SSc. The findings of this scoping review suggest that SSc-PVOD is characterized by a distinct clinical and microvascular profile, with some features that may help differentiate it from both idiopathic and hereditary PVOD and classic SSc-PAH.

Our literature review indicates that SSc-PVOD shares several clinical and demographic features with classic SSc-PAH, such as a predominantly older female population, limited cutaneous SSc subset, ACA positivity, and preserved lung volumes with impaired gas transfer. However, a markedly reduced DLCO (often < 30%) with elevated FVC/DLCO ratio, severe resting hypoxemia, and cardinal HRCT findings emerged as suggestive features. These characteristics may provide important clues for SSc-PVOD recognition in clinical practice. Importantly, approximately one-third of the patients included in our review developed pulmonary edema following vasodilator therapy for PAH, underscoring the persistent challenge of recognizing this condition in patients with scleroderma.

In contrast to hereditary PVOD, which is driven by biallelic loss-of-function mutations in the EIF2AK4 gene [4,33] and usually presents at a younger age, SSc-PVOD occurs in the context of an established autoimmune disease. This distinction may have implications for the applicability of existing diagnostic and prognostic algorithms for PVOD. For example, models developed by Ogawa et al. or Hadinnapola et al. incorporate male sex and extensive smoking histories as key weighting factors [34,35]. Because SSc-PVOD arises almost exclusively in non-smoking females with specific autoantibody profiles, these models cannot be generalized to CTD patients.

The distinct pathobiology of SSc-PVOD vs. idiopathic PVOD may further explain these differences. Unlike idiopathic forms, where acute or genetically driven loss of endothelial integrity plays a primary role, the chronic autoimmune environment in SSc, mediated by persistent endothelial activation, immune complex deposition, and dysregulated TGF-ꞵ signaling, induces progressive fibrous obliteration of the small pre-septal venules and septal veins [1,34]. A post-mortem study by Dorfmüller et al. and the recent histological series by Hurst et al. confirm that occlusive fibrous remodeling of septal veins and pre-septal venules (<100 µm in diameter) is present in up to 75% of CTD-associated pulmonary hypertension cases, supporting the hypothesis that post-capillary venular remodeling is an intrinsic component of scleroderma vasculopathy rather than an isolated anomaly [29,36].

This interpretation is consistent with the evolution of the classification of pulmonary hypertension. The 2022 ESC/ERS guidelines include “PAH with features of venous/capillary involvement” within Group 1 [2]: this concept may be particularly relevant in SSc, in which pulmonary vascular involvement occurs in the context of a systemic microangiopathy. Our findings therefore support the interpretation of SSc-PVOD as a phenotype of SSc-PAH characterized by prominent pulmonary venous and capillary involvement. In this scenario, the term “SSc-PAH with features of PVOD (SSc-PAH/PVOD)” may better capture the clinical and pathobiological overlap observed in SSc. Indeed, current evidence points toward a single unifying continuum of SSc-associated pulmonary vasculopathy, wherein “SSc-PVOD” persists primarily as legacy terminology for the high-risk, venous-prominent end of this spectrum rather than a separate biological entity. As previously highlighted by Nossent et al., PAH and PVOD in CTD represent extremes of a disease continuum rather than clear-cut dichotomy. Throughout this review, we maintained the term “SSc-PVOD” to be consistent with the legacy nomenclature used in the included source studies.

A notable finding emerging from our review is the particularly severe hemodynamic profile of SSc-PVOD, characterized by markedly elevated mPAP and PVR values, a pattern that is not consistently observed in SSc-PAH, particularly in settings where systematic screening programs are routinely implemented. When compared to classic SSc-PAH cohorts from major multicenter registries—such as the ASPIRE registry—patients with SSc-PVOD present with hemodynamic compromise that is equally severe or even worse [31]. In our experience, the rapid progression from a normal routine echocardiogram to a severe clinical phenotype may represent another clue suggesting PVOD. Notwithstanding these considerations, baseline RHC cannot distinguish SSc-PVOD from classic pre-capillary SSc-PAH, despite the striking severity of the hemodynamic profile observed in SSc-PVOD.

Because surgical lung biopsy carries prohibitively high morbidity and mortality in patients with severe pulmonary hypertension, the correct identification of PVOD relies largely on clinical and radiological features, such as the severely impaired gas exchange with hypoxemia and characteristic HRCT findings [10]. In our review we found that mechanical lung volumes (FVC and TLC) remain characteristically preserved, whereas diffusing capacity is profoundly and disproportionately collapsed, frequently dropping below 30% of predicted. While a reduced DLCO is common to both classic SSc-PAH and idiopathic PVOD cohorts described by Montani et al. and Hadinnapola et al., such a precipitous, isolated drop—accompanied by a markedly elevated FVC/DLCO ratio and resting hypoxemia—strongly points toward extensive capillary-venular obliteration, persistent microvascular congestion, and occult alveolar hemorrhage [13,34]. Evidence of the latter on bronchoalveolar lavage [37] or on non-induced sputum examination [38], characterized by an elevated proportion of hemosiderin-laden macrophages, may provide additional clues of pulmonary microvascular congestion and leakage. These features should be carefully interpreted in patients with SSc who may present with other disease-related cardiopulmonary involvement.

The classic radiological triad of centrilobular ground-glass opacities, smooth interlobular septal thickening, and mediastinal lymphadenopathy is not uniformly present, and its absence must not be used to rule out SSc-PVOD. While Günther et al. demonstrated that having two or more cardinal signs strongly predicts vasodilator-induced pulmonary edema, several patients in our pooled analysis exhibited only a single sign or completely lacked classic radiological abnormalities yet had histologically proven PVOD or developed acute pulmonary edema following vasodilator administration [8]. This is consisted with Hurst et al.’s findings: the sensitivity of the classic HRCT triad decreases to only 16.7% in patients where PVOD is superimposed on pre-existing fibrotic ILD, emphasizing that clinicians and pathologists must maintain high clinical suspicion, as the radiological features of PVOD may be less specific and more difficult to recognize in the presence of underlying parenchymal fibrosis [36]. Furthermore, non-specific centrilobular ground-glass opacities on HRCT are frequently misdiagnosed as early cellular alveolitis or active ILD [39], leading to inappropriate immunosuppressive therapy and delaying the diagnosis of pulmonary hypertension.

The therapeutic management of SSc-PVOD remains exceptionally challenging, as no medical therapy has demonstrated definitive clear benefits. In our pooled analysis, the majority of patients receiving PAH-targeted vasodilators developed acute pulmonary edema. Notably, while pulmonary vasodilators represent the cornerstone of treatment for classic Group 1 SSc-PAH, their use in SSc-PVOD requires caution due to the risk of acute pulmonary edema. The latest ESC/ERS guidelines suggest that PAH-targeted therapies may be considered in selected patients with concomitant PVOD features, with slow, low-dose titration and close clinical and hemodynamic monitoring. Diuretics and continuous supplemental oxygen to suppress hypoxic pulmonary vasoconstriction should be considered as supportive measures [33,40]. The high rate of pulmonary edema documented in our review (20/24 treated patients) largely reflects publication and selection bias inherent to retrospective series and case reports, which preferentially capture severe phenotypes. Consequently, the risk of pulmonary edema following vasodilators may be overestimated, and a cautious, case-by-case evaluation is required; in accordance with the latest guidelines [2], low-dose vasodilator monotherapy with slow titration may be considered in patients with milder phenotypes under strict monitoring in specialized centers.

Conventional immunosuppressive therapy fails to halt vascular obliteration in SSc-associated disease, although in a recent retrospective study, Finger et al. showed that incorporating moderate-dose prednisolone alongside vasodilators and tyrosine kinase inhibitors may improve survival in a small cohort of PVOD patients compared to non-steroid regimens, suggesting that targeting vascular and perivascular inflammation may stabilize capillary permeability and delay clinical decompensation while awaiting organ transplantation [41]. Even though Bergbaum et al. demonstrated functional stabilization, radiological regression, and hemodynamic improvement with mycophenolate mofetil and corticosteroids in three patients with idiopathic or EIF2AK4-related PVOD, their series excluded CTD-PVOD cases [42]. However, while all included patients had invasive confirmation of pre-capillary pulmonary hypertension with PAWP ≤15 mmHg and no evidence of primary left-sided heart disease, subtle left ventricular diastolic dysfunction or occult fluid shifts cannot be entirely excluded in cases diagnosed ex juvantibus, in contrast to those with gold-standard histological confirmation.

Emerging molecularly targeted strategies offer potential alternatives to classic vasodilators. Tyrosine kinase inhibition via imatinib has shown hemodynamic and functional stabilization in small clinical series. Although Ogawa et al. demonstrated that imatinib significantly reduced mPAP and extended median survival compared to untreated PVOD cohorts, largely by inhibiting platelet-derived growth factor (PDGF) signaling, their cohort included only one patient with SSc out of 16 subjects, substantially limiting the direct applicability of these findings to SSc-associated disease [43]. More importantly, the evaluation of novel disease-modifying agents with non-vasodilating mechanisms of action represents a crucial prospective avenue. In this context, sotatercept, a first-in-class activin receptor type IIA fusion protein that traps activins and growth differentiation factors to restore the balance between pro-proliferative TGF-β/activin signaling and anti-proliferative bone morphogenetic protein receptor type 2 (BMPR2) signaling, is of paramount interest. Unlike vasodilators, sotatercept acts primarily as an anti-remodeling agent [44]. By exerting its therapeutic effects directly on cellular proliferation and vascular reverse-remodeling without acutely increasing pre-capillary flow into an obstructed venous bed, sotatercept could theoretically circumvent the abrupt surge in transcapillary hydrostatic pressure that precipitates pulmonary edema. Recent reports by Aguado et al. describing hemodynamic improvement without pulmonary edema in patients with PVOD treated with sotatercept provide a preliminary biological rationale for exploring this pathway [45]. However, given the absence of dedicated cohort studies or clinical trials, further studies are required to confirm whether this anti-remodeling mechanism translates into real clinical benefit and safety in SSc-associated venular remodeling.

Bilateral lung transplantation remains the only definitive, life-saving treatment, offering 5-year post-transplant survival rates (73–81%) comparable to idiopathic PAH as documented by Montani et al. and Lechartier et al. [41,46]. Both PVOD and SSc-PAH are considered high-risk phenotypes; therefore, their coexistence should prompt early referral to a specialized lung transplantation center [2,47]. Nevertheless, transplant eligibility in SSc is severely constrained by multisystem extrapulmonary disease, representing a major hurdle not typically encountered in younger patients with idiopathic or heritable PVOD [1,2]. As outlined in the International Society for Heart and Lung Transplantation (ISHLT) consensus document [47], severe gastrointestinal involvement—particularly advanced esophageal dysmotility and gastroesophageal reflux—markedly increases the risk of recurrent allograft microaspiration, chronic lung allograft dysfunction (CLAD), and bronchiolitis obliterans syndrome. Furthermore, renal impairment, primary scleroderma-related myocardial fibrosis, peripheral digital ischemia and restrictive chest wall tethering secondary to extensive cutaneous sclerosis frequently constitute relative or absolute contraindications, contributing to the substantial proportion of ineligible candidates and the elevated waitlist mortality reported in SSc cohorts.

Our critical analysis of the literature reveals several major methodological limitations and knowledge gaps. The available evidence base is predominantly composed of retrospective single-center case reports, small series, and registry sub-analyses. The lack of individual patient data (IPD) across multi-institutional registries precludes multivariable risk adjustment, propensity score matching, and formal meta-analyses. Additionally, publication bias is evident, as cases complicated by catastrophic pulmonary edema or fatal outcomes are disproportionately reported compared to indolent phenotypes. Furthermore, a structural boundary of the available literature is the near-universal exclusion of patients with substantial fibrotic ILD across primary cohorts and case series, implemented to rule out Group 3 pulmonary hypertension. Consequently, the findings synthesized in this review reflect a pure vascular phenotype and cannot be directly extrapolated to patients with overlapping SSc-ILD and PVOD. In clinical practice, concomitant parenchymal fibrosis is frequent, and as shown by Hurst et al., the diagnostic sensitivity of the classic HRCT triad drops to 16.7% when venular remodeling occurs within fibrotic parenchyma [36]. The persistent lack of studies characterizing this overlapping SSc-ILD/PVOD subgroup represents a critical evidence gap that warrants dedicated prospective investigation.

The major strength of this scoping review is its comprehensive integration of multi-domain parameters, spanning clinical, serological, functional, hemodynamic, radiological, and histopathological criteria, across all published cases of SSc-PVOD. Its primary limitation is the unavoidable reliance on aggregate descriptive data from retrospective cohorts with variable diagnostic criteria (histological vs. radiological vs. ex juvantibus clinical diagnoses). To resolve these dilemmas, future research must focus on establishing prospective, multicenter international registries dedicated to CTD-associated pulmonary vascular diseases. By standardizing IPD collection, centralizing HRCT and RHC data, and prospectively evaluating composite non-invasive markers (including ACA status, FVC/DLCO ratios, and HRCT cardinal signs), collaborative consortia can validate this high-risk clinical profile, optimize the timing of lung transplant referral, and evaluate novel anti-remodeling therapies, such as sotatercept, in a controlled setting.

## 5. Conclusions

PVOD represents a distinct, life-threatening form of pulmonary vascular involvement in SSc. Predominantly affecting older females with limited cutaneous disease and anti-centromere antibodies, SSc-PVOD presents with a markedly severe pre-capillary hemodynamic profile that is indistinguishable from that of SSc-PAH. Consequently, recognizing a non-invasive high-risk profile—integrating severe, isolated DLCO impairment, disproportionate hypoxemia, and the cardinal thoracic HRCT signs—is essential to guide diagnosis and prevent vasodilator-induced pulmonary edema. This challenge is particularly relevant in patients with SSc, in whom venular involvement may complicate PAH, potentially representing a continuum of scleroderma-associated pulmonary microangiopathy. In the absence of established curative medical therapies, prompt evaluation for bilateral lung transplantation remains the primary life-saving strategy in eligible candidates. Prospective multicenter registries with standardized patient-level data are needed to identify non-invasive markers of venular involvement and develop predictive models to detect SSc-PAH patients at high risk of underlying PVOD before pulmonary vasodilator therapy, while supporting the evaluation of novel anti-remodeling therapies, such as activin signaling inhibitors, which may stabilize vascular remodeling without inducing hydrostatic alveolar flooding.

## Figures and Tables

**Table 1:** Study design and diagnostic criteria for pulmonary veno-occlusive disease across included studies.

		HRCT PVOD Diagnostic Criteria		
Author (Year)	Study Design	Histological Appearance	≥2 Chest HRCT PVOD Findings	Poor Response to Vasodilators
N = 20		N = 8	N = 12	N = 9
Connolly, M.J. et al. (2017) [10]	Retrospective Cohort		X	
Daraban, A.M. et al. (2015) [16]	Case Report		X	X
Diao, X.-L., Mu, X.-D. and Jin, M.-L. (2017) [17]	Case Report	X		
Dorfmüller, P. et al. (2007) [29]	Retrospective Case-Control	X		
Duarte, A.C. et al. (2020) [9]	Case Report		X	X
Fujisaki, S. et al. (2026) [32]	Retrospective Cohort + Autoptic Case Series	X		
Garg, S., Gupta, D. and Sindwani, G. (2025) [18]	Case Report		X	X
Gugnani, M.K. et al. (2000) [19]	Case Report		X	X
Günther, S. et al. (2012) [8]	Retrospective Cohort + Autoptic Case Series		X	
Jeevagan, A. (2010) [20]	Case Report	X		
Johnson, S.R. et al. (2006) [27]	Case Series	X	X	
Kyono, M. et al. (2020) [21]	Case Report			X
McGuire, F. et al. (2010) [22]	Case Report			X
Nakamura, J. et al. (2023) [28]	Case Series		X	
Reddy, T.P. et al. (2024) [30]	Case Report	X		X
Saito, A. et al. (2003) [23]	Case Report	X	X	
Sidda, N. et al. (2025) [24]	Case Report		X	X
Smith, H. et al. (2024) [31]	Retrospective Cohort		X	
Tada, H. et al. (2012) [25]	Case Report	X		X
Van Leeuwen, N.M. et al. (2020) [26]	Case Report		X	

Summary of the 20 included publications detailing study design and the diagnostic criteria applied to establish pulmonary veno-occlusive disease (PVOD) or pulmonary capillary hemangiomatosis (PCH), categorized into histological confirmation, presence of >2 cardinal high-resolution computed tomography (HRCT) features, and poor response or development of acute pulmonary edema following targeted vasodilator therapy. Abbreviations: HRCT, high-resolution computed tomography; N, number of studies/cases; PCH, pulmonary capillary hemangiomatosis; PVOD, pulmonary veno-occlusive disease.

**Table 2:** Demographic characteristics, systemic sclerosis cutaneous subtypes, and autoantibody profiles of the study population.

			Sex (N = 47)		SSc Subtype (N = 43)		Autoantibody Positivity (N = 14)						
Study Author (Year)	Population	Age	Female	Male	lcSSc	dcSSc	ACA	Anti-Fibrillarin	Anti-RNA Pol	Anti-Ro	Anti-U1-RNP	Anti-Scl70	Anti-Th/To
Total	63		41	6	40	3	13	1	1	1	1	0	1
Connolly, M.J. et al. (2017) [10]	7	59 ± 9	6	1	7	0	5	1	1	0	0	0	1
Daraban, A.M. et al. (2015) [16]	1	48	1	0	0	0	NR	NR	NR	NR	NR	NR	NR
Diao, X.-L., Mu, X.-D. and Jin, M.-L. (2017) [17]	1	65	1	0	1	0	1	0	0	0	0	0	0
Dorfmüller, P. et al. (2007) [29]	4	66 ± 8	2	2	0	0	NR	NR	NR	NR	NR	NR	NR
Duarte, A.C. et al. (2020) [9]	1	72	1	0	0	0	1	0	0	0	0	0	0
Fujisaki, S. et al. (2026) [32]	1	55	NR	0	NR	NR	NR	NR	NR	NR	NR	NR	NR
Garg, S., Gupta, D. and Sindwani, G. (2025) [18]	1	55	1	0	1	0	1	0	0	0	0	0	0
Gugnani, M.K. et al. (2000) [19]	1	60	1	0	1	0	1	0	0	0	0	0	0
Günther, S. et al. (2012) [8]	16	66	NR	NR	13	3	NR	NR	NR	NR	NR	NR	NR
Jeevagan, A. (2010) [20]	1	NR	1	0	0	0	1	0	0	0	1	0	0
Johnson, S.R. et al. (2006) [27]	4	75	4	NR	0	0	1	0	0	NR	0	0	NR
Kyono, M. et al. (2020) [21]	1	53	1	0	NR	NR	1	0	NR	1	0	0	NR
McGuire, F. et al. (2010) [22]	1	52	1	0	1	0	NR	NR	NR	NR	NR	NR	NR
Nakamura, J. et al. (2023) [28]	2	64 ± 22	2	0	1	0	NR	NR	NR	NR	NR	NR	NR
Reddy, T.P. et al. (2024) [30]	1	52	1	0	0	NR	NR	NR	NR	NR	NR	NR	NR
Saito, A. et al. (2003) [23]	1	70	0	0	NR	NR	0	0	0	0	0	0	0
Sidda, N. et al. (2025) [24]	1	45	1	0	0	NR	0	0	0	0	0	0	0
Smith, H. et al. (2024) [31]	17	66 ± 12	14	3	1	0	NR	NR	NR	NR	NR	NR	NR
Tada, H. et al. (2012) [25]	1	55	1	0	1	0	NR	NR	0	0	NR	NR	NR
Van Leeuwen, N.M. et al. (2020) [26]	1	45	1	0	1	0	1	0	0	0	0	0	0

Baseline demographic parameters (age, sex distribution), clinical cutaneous classification of systemic sclerosis (limited vs. diffuse cutaneous disease), and serum autoantibody profiles characterized across the aggregated patient cohort. Abbreviations: ACA, anti-centromere antibodies; Anti-RNA pol, anti-RNA polymerase III antibodies; Anti-Ro, anti-Ro/SSA antibodies; Anti-Scl70, anti-topoisomerase I antibodies; Anti-Th/To, anti-Th/To ribonucleoprotein antibodies; Anti-U1-RNP, anti-U1 ribonucleoprotein antibodies; dcSSc, diffuse cutaneous systemic sclerosis; lcSSc, limited cutaneous systemic sclerosis; N, number of patients; NR, not reported; SSc, systemic sclerosis.

**Table 3:** High-resolution computed tomography morphological alterations and prevalence of cardinal signs of PVOD.

		Chest CT Alterations Reported (N = 19)										PVOD HRCT Cardinal Signs (N = 54)			
Study Author (Year)	Population	Ground Glass	Centrilobular GGO	Diffuse GGO	Patchy GGO	Mosaic Perfusion	Interlobular Septal Thickening	Mediastinal Lymphadenopathy	Fibrosis	Pleural Effusion	PA Dilatation	None	1	2	3
Total	63	9	1	4	2	1	5	7	1	4	8	3	5	45	1
Connolly, M.J. et al. (2017) [10]	7	NR	NR	NR	NR	NR	NR	NR	NR	NR	NR	0	0	7	0
Daraban, A.M. et al. (2015) [16]	1	1	1	0	0	NR	1	1	1	1	1	0	0	0	1
Diao, X.-L., Mu, X.-D. and Jin, M.-L. (2017) [17]	1	0	1	NR	0	0	0	0	0	0	1	0	1	0	0
Dorfmüller, P. et al. (2007) [29]	4	NR	NR	NR	NR	NR	NR	NR	NR	NR	NR	NR	NR	NR	NR
Duarte, A.C. et al. (2020) [9]	1	1	0	1	0	NR	0	1	0	1	0	0	1	0	0
Fujisaki, S. et al. (2026) [32]	1	NR	NR	NR	NR	NR	NR	NR	NR	NR	NR	NR	NR	NR	NR
Garg, S., Gupta, D. and Sindwani, G. (2025) [18]	1	NR	NR	NR	NR	0	NR	NR	NR	0	0	0	0	0	0
Gugnani, M.K. et al. (2000) [19]	1	0	0	0	0	NR	0	0	0	NR	NR	1	0	0	0
Günther, S. et al. (2012) [8]	16	NR	NR	NR	NR	NR	NR	NR	NR	NR	NR	0	0	16	0
Jeevagan, A. (2010) [20]	1	0	0	0	NR	NR	0	0	0	NR	1	1	0	0	0
Johnson, S.R. et al. (2006) [27]	4	3	0	1	1	0	2	3	0	2	2	0	1	2	0
Kyono, M. et al. (2020) [21]	1	0	0	0	NR	1	0	NR	NR	0	1	1	0	0	0
McGuire, F. et al. (2010) [22]	1	1	0	1	NR	NR	NR	1	0	NR	1	0	1	0	0
Nakamura, J. et al. (2023) [28]	2	NR	NR	NR	NR	NR	NR	0	0	0	NR	0	1	2	0
Reddy, T.P. et al. (2024) [30]	1	NR	NR	NR	NR	NR	NR	NR	NR	NR	NR	0	0	0	0
Saito, A. et al. (2003) [23]	1	1	0	0	1	NR	1	NR	NR	NR	1	0	1	0	0
Sidda, N. et al. (2025) [24]	1	NR	NR	NR	NR	NR	NR	NR	NR	NR	NR	0	0	0	0
Smith, H. et al. (2024) [31]	17	NR	NR	NR	NR	NR	0	NR	NR	NR	NR	0	0	17	0
Tada, H. et al. (2012) [25]	1	NR	NR	NR	NR	NR	NR	1	0	NR	NR	0	0	0	0
Van Leeuwen, N.M. et al. (2020) [26]	1	1	0	0	0	0	1	1	0	0	1	0	0	1	0

**Table 4:** Hemodynamic parameters from right heart catheterization and pulmonary function test characteristics.

		Right Heart Catheterization (N = 57)				Pulmonary Function Test (N = 59)									
Study Author (Year)	Population (n)	mPAP (mmHg)	PAWP (mmHg)	CO (L/min)	PVR (WU)	FEV1%	FVC L	FVC %	FEV1/FVC%	TLC%	DLCO%	DLCO >30%	DLCO <30%	FVC/DLCO %	6MWT (m)
Total	63	57	52	44	54	48	28	29	27	29	48	13	12	9	29
Connolly, M.J. et al. (2017) [10]	7	38 ± 8	11 ± 3	NR	6.44 ± 2.35	75 ± 19	NR	84 ± 23	66 ± 13	92 ± 17	41 ± 18	NR	NR	NR	246 ± 154
Daraban, A.M. et al. (2015) [16]	1	42	11	NR	6.27	82.7	2.44	85.1	85	104.6	27	0	1	3.15	336
Diao, X.-L., Mu, X.-D. and Jin, M.-L. (2017) [17]	1	NR	NR	NR	NR	NR	NR	NR	NR	NR	NR	NR	NR	NR	NR
Dorfmüller, P. et al. (2007) [29]	4	51 ± 3	NR	NR	27.2 ± 10.8	NR	NR	NR	89 ± 7	83 ± 10	NR	NR	NR	NR	NR
Duarte, A.C. et al. (2020) [9]	1	44	8	NR	11.5	NR	NR	105	NR	NR	45	1	0	NR	NR
Fujisaki, S. et al. (2026) [32]	1	44	7	NR	20	79	NR	96	82	NR	62.9	1	0	1.53	550
Garg, S., Gupta, D. and Sindwani, G. (2025) [18]	1	NR	NR	NR	NR	NR	NR	NR	NR	NR	40	0	1	NR	240
Gugnani, M.K. et al. (2000) [19]	1	NR	NR	NR	NR	NR	NR	91	70	102	50	1	0	1.82	NR
Günther, S. et al. (2012) [8]	16	48 ± 10	7 ± 3	4.6 ± 1.3	9.8 ± 4.2	87 ± 23	NR	NR	NR	91 ± 19	34 ± 9	NR	NR	NR	294 ± 85
Jeevagan, A. (2010) [20]	1	NR	NR	NR	NR	NR	NR	NR	NR	NR	NR	2	1	NR	NR
Johnson, S.R. et al. (2006) [27]	4	39.5	12	4.2	NR	90	3.01	86.5	80	NR	35	0	1	2.81	NR
Kyono, M. et al. (2020) [21]	1	26	5	2.1	6.2	73.6	NR	80.4	NR	NR	17	1	0	5.36	333
McGuire, F. et al. (2010) [22]	1	72	NR	NR	NR	90	3.29	90	78	NR	40	1	0	2.25	NR
Nakamura, J. et al. (2023) [28]	2	38 ± 1	6 ± 2	3.7 ± 0.5	9.0 ± 1.3	49	2.05 ± 0.69	81 ± 44	79	NR	29 ± 10	0	1	NR	NR
Reddy, T.P. et al. (2024) [30]	1	NR	NR	7	NR	NR	NR	83.2	NR	NR	52.3	NR	NR	1.59	359
Saito, A. et al. (2003) [23]	1	NR	5	NR	NR	NR	NR	NR	NR	NR	20	NR	NR	NR	NR
Sidda, N. et al. (2025) [24]	1	NR	NR	NR	NR	NR	NR	NR	NR	NR	NR	NR	NR	NR	NR
Smith, H. et al. (2024) [31]	17	49.1 ± 9.4	9.4 ± 3	3.9 ± 1.8	12	94.5 ± 15	NR	108.1 ± 12	73.2 ± 10	NR	34.7 ± 8	NR	NR	NR	NR
Tada, H. et al. (2012) [25]	1	48	14	3.9	6.75	NR	NR	NR	NR	NR	NR	NR	NR	NR	NR
Van Leeuwen, N.M. et al. (2020) [26]	1	39	13	8	3.3	NR	NR	NR	NR	NR	38	1	0	NR	123

Invasive hemodynamic measurements obtained via right heart catheterization (mean pulmonary arterial pressure, pulmonary arterial wedge pressure, cardiac output, and pulmonary vascular resistance) alongside resting pulmonary function metrics (forced expiratory volume in 1 s, forced vital capacity, total lung capacity, diffusing capacity for carbon monoxide, FVC/DLCO ratio) and exercise capacity assessed via the 6 min walk distance. Abbreviations: 6MWT, 6 min walk test distance (meters); CO, cardiac output (liters per minute); DLCO, diffusing capacity of the lung for carbon.

**Table 5:** Major clinical complications following vasodilator exposure, lung transplantation status, and causes of mortality.

		Major Complication (N = 21)				Number of Deaths (N = 33)					PAH Treatment		
Study Author (Year)	Population	Pulm Edema	Acute RHF	Lung Transplant	Lung Transplant List	Not Reported	Right Heart Failure	Cardiopulmonary Arrest	GI Bleeding	Renal Failure	ERA	PDE5I	Prostanoids
Total	63	20	3	2	5	27	2	2	1	1	9	6	7
Connolly, M.J. et al. (2017) [10]	7	2	0	NR	NR	5	NR	NR	NR	NR	NR	NR	1
Daraban, A.M. et al. (2015) [16]	1	1	0	0	1	0	0	0	0	0	1	0	0
Diao, X.-L., Mu, X.-D. and Jin, M.-L. (2017) [17]	1	0	0	1	1	0	0	0	0	0	1	0	0
Dorfmüller, P. et al. (2007) [29]	4	2	0	1	1	3	NR	NR	NR	NR	NR	NR	NR
Duarte, A.C. et al. (2020) [9]	1	1	0	0	0	0	0	0	0	0	1	1	0
Fujisaki, S. et al. (2026) [32]	1	NR	NR	NR	NR	1	NR	NR	NR	NR	NR	NR	NR
Garg, S., Gupta, D. and Sindwani, G. (2025) [18]	1	1	1	0	0	0	0	0	0	0	1	1	0
Gugnani, M.K. et al. (2000) [19]	1	1	1	0	NR	1	1	0	0	0	0	0	1
Günther, S. et al. (2012) [8]	16	8	0	NR	0	14	NR	NR	NR	NR	NR	NR	NR
Jeevagan, A. (2010) [20]	1	0	0	0	0	1	0	1	0	0	0	0	0
Johnson, S.R. et al. (2006) [27]	4	1	0	0	1	4	NR	NR	NR	NR	2	0	2
Kyono, M. et al. (2020) [21]	1	0	0	0	0	0	0	0	0	0	0	1	0
McGuire, F. et al. (2010) [22]	1	1	0	0	0	1	0	1	0	0	0	0	1
Nakamura, J. et al. (2023) [28]	2	0	0	0	0	0	NR	NR	0	0	NR	NR	NR
Reddy, T.P. et al. (2024) [30]	1	0	1	0	NR	1	1	0	NR	NR	1	0	1
Saito, A. et al. (2003) [23]	1	NR	0	0	0	1	0	0	1	0	NR	NR	NR
Sidda, N. et al. (2025) [24]	1	1	NR	NR	0	0	0	0	0	0	0	1	0
Smith, H. et al. (2024) [31]	17	NR	0	0	0	0	0	0	0	0	NR	NR	NR
Tada, H. et al. (2012) [25]	1	1	0	0	1	1	0	0	0	1	1	1	1
Van Leeuwen, N.M. et al. (2020) [26]	1	0	0	0	0	0	0	0	0	0	1	1	0

Summary of major treatment-related adverse events (iatrogenic acute pulmonary edema and acute right heart failure), and definitive lung transplantation and active waitlist status, along with overall all-cause mortality and specific primary causes of death identified across the cumulative cohort of 63 patients. Abbreviations: GI, gastrointestinal; N, number of patients; NR, not reported; RHF, right heart failure; PAH, pulmonary arterial hypertension; ERA, endothelin receptor antagonists; PDE5I, phosphodiesterase type 5 inhibitor.

## Data Availability

The data underlying this article will be shared upon reasonable request to the corresponding author.

## Supplementary Materials

The following supporting information can be downloaded at https://www.mdpi.com/article/10.3390/jcdd13100506/s1, Supplementary Material S1: PRISMA-ScR-PVOD-SSc-Checklist; Supplementary Material S2: Study Protocol PVOD-SSc.
