# Supplementary material for: Pulmonary Veno-Occlusive Disease in Systemic Sclerosis: A Scoping Review of the Literature
Source: J Cardiovasc Dev Dis. 2026 Oct 8;13(10):506. doi: 10.3390/jcdd13100506 (PMC13705322; doi:10.3390/jcdd13100506)
Supplement: Supplementary file 1 [file jcdd-13-00506-s001.zip › S2-Study Protocol PVOD-SSc.pdf]

Study Protocol: Pulmonary veno-occlusive disease in systemic sclerosis - a scoping review of the literature

Francesco Carta[1], Beatrice Moccaldi[1], Elisabetta Zanatta[1,2]

[1] Rheumatology Unit, Department of Medicine-DIMED, Padova University Hospital, 35128 Padova, Italy
[2] Department of Cardiac, Thoracic, Vascular Sciences and Public Health, Padova University Hospital, 35128 Padova, Italy

Registered on Open Science Framework: DOI 10.17605/OSF.IO/TR8H5

1. Background & Rationale

Systemic sclerosis (SSc) is a complex autoimmune connective tissue disease characterized by immune dysregulation, widespread microvascular damage, and progressive tissue fibrosis. Among SSc complications, pulmonary arterial hypertension (PAH) represents a leading cause of morbidity and mortality. Pulmonary veno-occlusive disease (PVOD) and pulmonary capillary hemangiomatosis (PCH)—grouped together under Group 1 pulmonary hypertension in the updated European Society of Cardiology / European Respiratory Society (ESC/ERS) classification—represent a rare, life-threatening, and underrecognized entity.

Pathologically, PVOD is defined by fibrous, obliterative remodeling of the post-capillary pulmonary venules. Clinically and hemodynamically, SSc-PVOD closely mimics classic pre-capillary SSc-PAH, presenting with severe pre-capillary pulmonary hypertension on right heart catheterization (RHC) with normal pulmonary arterial wedge pressure (PAWP). However, administering standard PAH vasodilators can trigger fatal hydrostatic pulmonary edema. Despite its prognostic implications, existing evidence is dispersed across case reports and retrospective cohorts with significant clinical heterogeneity. This scoping review protocol is designed to systematically map the literature, define clinical/diagnostic features, identify knowledge gaps, and guide future prospective registries.

2. Review Question & PCC Framework

This scoping review is guided by the Population, Concept, and Context (PCC) framework:

- Population (P): Adult patients ($\geq$ 18 years) diagnosed with systemic sclerosis presenting with right heart catheterization-confirmed pre-capillary pulmonary hypertension.
- Concept (C): A concurrent or subsequent confirmed diagnosis of pulmonary veno-occlusive disease (PVOD) or pulmonary capillary hemangiomatosis (PCH).
- Context (C): Inpatient, outpatient, or tertiary referral settings globally reporting diagnostic, functional, hemodynamic, therapeutic, or outcome parameters.

3. Eligibility Criteria

Inclusion Criteria

- Adult patients ($\geq$ 18 years) with systemic sclerosis.
- Pre-capillary pulmonary hypertension confirmed by RHC according to updated criteria: mean pulmonary arterial pressure (mPAP) $>$ 20 mmHg, PAWP $\leq$ 15 mmHg, and pulmonary vascular resistance (PVR) $>$ 2.0 Wood Units.
- Diagnosis of PVOD or PCH established via at least one of the following:

1. Histopathological confirmation (venous luminal narrowing, intimal fibrosis, alveolar capillary multiplication);

2. High-resolution computed tomography (HRCT) displaying ≥ 2 cardinal signs (centrilobular ground-glass opacities, smooth interlobular septal thickening, mediastinal lymphadenopathy);

3. Ex-juvantibus/clinical diagnosis characterized by acute pulmonary edema or severe deterioration following pulmonary vasodilator initiation.

- Availability of extractable demographic, clinical, hemodynamic, or radiological data.
- Study designs: observational cohort studies, case-control studies, case series, and individual case reports.

Exclusion Criteria

- Presence of significant interstitial lung disease (ILD) or extensive parenchymal fibrosis on HRCT.
- Moderately severe to very severe obstructive pattern on pulmonary function tests (FEV1 < 60% predicted with FEV1/FVC < 70%).
- Restrictive ventilatory defect defined by Total Lung Capacity below the lower limit of normal (TLC < LLN).
- Secondary, non-Group 1 pulmonary hypertension etiologies (e.g., left heart disease, thromboembolic disease).
- Pediatric or juvenile SSc cases.
- Preclinical, animal, or *in vitro* experimental studies.
- Conference abstracts without accessible full text, editorials, and non-English publications.

4. Information Sources & Search Strategy

A comprehensive systematic literature search is conducted across PubMed/MEDLINE, Scopus, and the Cochrane Library covering the period from January 1970 to May 2026 (final search rerun: May 20, 2026).

The search utilizes Medical Subject Headings (MeSH) and free-text terms in Title/Abstract using Boolean logic (AND, OR):

- Pubmed: ("pulmonary veno-occlusive disease"[MeSH Terms] OR "pulmonary veno-occlusive disease"[tiab] OR "PVOD"[tiab] OR "pulmonary capillary hemangiomatosis"[tiab] OR "PCH"[tiab] OR "pulmonary venous involvement"[tiab] OR "pulmonary venous hypertension"[tiab] OR "postcapillary vasculopathy"[tiab] OR "venous remodeling"[tiab]) AND ("systemic sclerosis"[MeSH Terms] OR "systemic sclerosis"[tiab] OR "scleroderma"[tiab] OR "SSc"[tiab] OR "CREST syndrome"[tiab] OR "limited cutaneous systemic sclerosis"[tiab] OR "lcSSc"[tiab] OR "diffuse cutaneous systemic sclerosis"[tiab] OR "dcSSc"[tiab] OR "connective tissue disease"[tiab] OR "CTD"[tiab]);
- Scopus: (TITLE-ABS-KEY ("pulmonary veno-occlusive disease") OR TITLE-ABS ("PVOD") OR TITLE-ABS ("pulmonary capillary hemangiomatosis") OR TITLE-ABS ("PCH") OR TITLE-ABS ("pulmonary venous involvement") OR TITLE-ABS ("pulmonary venous hypertension") OR TITLE-ABS ("postcapillary vasculopathy") OR TITLE-ABS ("venous remodeling")) AND (TITLE-ABS-KEY ("systemic sclerosis") OR TITLE-ABS ("scleroderma") OR TITLE-ABS ("SSc") OR TITLE-ABS ("CREST syndrome") OR TITLE-ABS ("limited cutaneous systemic sclerosis") OR TITLE-ABS ("lcSSc") OR TITLE-ABS ("diffuse cutaneous systemic sclerosis") OR TITLE-ABS ("dcSSc") OR TITLE-ABS ("connective tissue disease") OR TITLE-ABS ("CTD"));

- Cochrane: ((dcSSc OR connective tissue disease OR CTD OR CREST syndrome OR limited cutaneous systemic sclerosis OR lcSSc OR diffuse cutaneous systemic sclerosis OR Scleroderma, Systemic OR systemic sclerosis OR scleroderma OR SSc) AND (venous remodeling OR PCH OR pulmonary venous involvement OR pulmonary venous hypertension OR postcapillary vasculopathy OR Hypertension, Pulmonary OR pulmonary veno-occlusive disease OR PVOD OR pulmonary capillary hemangiomatosis)).

5. Study Selection & Screening Process

Search records are imported into the Rayyan web platform for duplicate removal and multi-reviewer screening. Title and abstract screening, followed by full-text eligibility assessment, is performed independently by two investigators (FC, BM). Any discrepancies or disagreements are resolved through discussion and consensus. Study selection flow is recorded according to the PRISMA-ScR flowchart.

6. Data Extraction Plan

Data charting is carried out independently using a standardized data extraction template. Extracted variables comprise:

- Study Characteristics: Authors, publication year, country, study design.
- Demographics & SSc Profile: Age, sex, SSc cutaneous subtype (lcSSc vs. dcSSc), serum autoantibody profile (ACA, Anti-Scl70, Anti-RNA pol III, etc.).
- HRCT Imaging Features: Ground-glass opacities (centrilobular, patchy, diffuse), smooth interlobular septal thickening, mediastinal lymphadenopathy, PA dilatation, pleural effusion, and cardinal sign count (≥ 2 signs).
- Invasive Hemodynamics (RHC): mPAP, PAWP, cardiac output (CO), pulmonary vascular resistance (PVR).
- Pulmonary Function Tests & Exercise: FVC, FEV1, FEV1/FVC ratio, TLC, DLCO, FVC/DLCO ratio, 6-minute walk distance (6MWD).
- Histopathology: Post-mortem or surgical findings (venous occlusion, capillary proliferation, arteriolar changes).
- Treatments & Outcomes: Exposure to PAH vasodilators (ERAs, PDE5i, prostanoids), development of iatrogenic pulmonary edema, right heart failure, lung transplant status, and all-cause mortality.

7. Data Synthesis & Mapping

Because of expected heterogeneity across case reports, case series, and registry sub-analyses, individual patient data (IPD) meta-analysis cannot be performed. Data synthesis is structured as a comprehensive thematic mapping and qualitative narrative synthesis adhering to the PRISMA-ScR guidelines. Continuous variables are summarized as means ± standard deviation or medians with ranges, while categorical variables are reported as frequencies and percentages.

References

1. Denton, C.P.; Khanna, D. Systemic sclerosis. *Lancet* **2017**, *390*, 1685–1699. https://doi.org/10.1016/S0140-6736(17)30933-9.

2. Humbert, M.; Kovacs, G.; Hoeper, M.M.; Badagliacca, R.; Berger, R.M.F.; Brida, M.; Carlsen, J.; Coats, A.J.S.; Escribano-Subias, P.; Ferrari, P.; et al. 2022 ESC/ERS Guidelines for the diagnosis and treatment of pulmonary hypertension. *Eur. Respir. J.* **2023**, *61*, 2200879. https://doi.org/10.1183/13993003.00879-2022.

3. Montani, D.; Girerd, B.; Jaïs, X.; Levy, M.; Amar, D.; Savale, L.; Dorfmüller, P.; Seferian, A.; Lau, E.M.; Eyries, M.; et al. Clinical phenotypes and outcomes of heritable and sporadic pulmonary veno-occlusive disease: A population-based study. *Lancet Respir. Med.* **2017**, *5*, 125–134. https://doi.org/10.1016/S2213-2600(16)30438-6.

4. Eyries, M.; Montani, D.; Girerd, B.; Perret, C.; Leroy, A.; Lonjou, C.; Chelghoum, N.; Coulet, F.; Bonnet, D.; Dorfmüller, P.; et al. EIF2AK4 mutations cause pulmonary veno-occlusive disease, a recessive form of pulmonary hypertension. *Nat. Genet.* **2014**, *46*, 65–69. https://doi.org/10.1038/ng.2844.

5. Weatherald, J.; Dorfmüller, P.; Perros, F.; Ghigna, M.-R.; Girerd, B.; Humbert, M.; Montani, D. Pulmonary capillary haemangiomatosis: a distinct entity? *Eur. Respir. Rev.* **2020**, *29*, 190168. https://doi.org/10.1183/16000617.0168-2019.

6. Montani, D.; Lau, E.M.; Dorfmüller, P.; Girerd, B.; Jaïs, X.; Savale, L.; Perros, F.; Nossent, E.; Garcia, G.; Parent, F.; et al. Pulmonary veno-occlusive disease. *Eur. Respir. J.* **2016**, *47*, 1518–1534. https://doi.org/10.1183/13993003.00026-2016.

7. Miura, A.; Akagi, S.; Nakamura, K.; Ohta-Ogo, K.; Hashimoto, K.; Nagase, S.; Kohno, K.; Kusano, K.; Ogawa, A.; Matsubara, H.; et al. Different sizes of centrilobular ground-glass opacities in chest high-resolution computed tomography of patients with pulmonary veno-occlusive disease and patients with pulmonary capillary hemangiomatosis. *Cardiovasc. Pathol.* **2013**, *22*, 287–293. https://doi.org/10.1016/j.carpath.2012.12.002.

8. Günther, S.; Jaïs, X.; Maitre, S.; Bérezné, A.; Dorfmüller, P.; Seferian, A.; Savale, L.; Mercier, O.; Fadel, E.; Sitbon, O.; et al. Computed tomography findings of pulmonary venoocclusive disease in scleroderma patients presenting with precapillary pulmonary hypertension. *Arthritis Rheum.* **2012**, *64*, 2995–3005. https://doi.org/10.1002/art.34501.

9. Duarte, A.C.; Cordeiro, A.; Loureiro, M.J.; Ferreira, F. Pulmonary veno-occlusive disease: A probably underdiagnosed cause of pulmonary hypertension in systemic sclerosis. *Clin. Rheumatol.* **2020**, *39*, 1687–1691. https://doi.org/10.1007/s10067-020-04953-4.

10. Connolly, M.J.; Abdullah, S.; Ridout, D.A.; Schreiber, B.E.; Haddock, J.A.; Coghlan, J.G. Prognostic significance of computed tomography criteria for pulmonary veno-occlusive disease in systemic sclerosis-pulmonary arterial hypertension. *Rheumatology* **2017**, *56*, 2197–2203. https://doi.org/10.1093/rheumatology/kex351.

11. Tricco, A.C.; Lillie, E.; Zarin, W.; O'Brien, K.K.; Colquhoun, H.; Levac, D.; Moher, D.; Peters, M.D.J.; Horsley, T.; Weeks, L.; et al. PRISMA Extension for Scoping Reviews (PRISMA-ScR): Checklist and Explanation. *Ann. Intern. Med.* **2018**, *169*, 467–473. https://doi.org/10.7326/M18-0850.

12. Stewart, L.A.; Clarke, M.; Rovers, M.; Riley, R.D.; Simmonds, M.; Stewart, G.; Tierney, J.F.; PRISMA-IPD Development Group. Preferred Reporting Items for a Systematic Review and Meta-analysis of Individual Participant Data: The PRISMA-IPD Statement. *JAMA* **2015**, *313*, 1657–1665. https://doi.org/10.1001/jama.2015.3656.

13. Montani, D.; Price, L.C.; Dorfmuller, P.; Achouh, L.; Jaïs, X.; Yaïci, A.; Sitbon, O.; Musset, D.; Simonneau, G.; Humbert, M. Pulmonary veno-occlusive disease. *Eur. Respir. J.* **2009**, *33*, 189–200. https://doi.org/10.1183/09031936.00090608.

14. Goh, N.S.L.; Desai, S.R.; Veeraraghavan, S.; Hansell, D.M.; Copley, S.J.; Maher, T.M.; Corte, T.J.; Sander, C.R.; Ratoff, J.; Devaraj, A.; et al. Interstitial Lung Disease in Systemic Sclerosis. *Am. J. Respir. Crit. Care Med.* **2008**, *177*, 1248–1254. https://doi.org/10.1164/rccm.200706-877OC.

15. Ouzzani, M.; Hammady, H.; Fedorowicz, Z.; Elmagarmid, A. Rayyan—A web and mobile app for systematic reviews. *Syst. Rev.* **2016**, *5*, 210. https://doi.org/10.1186/s13643-016-0384-4.
